# Intraoperative cytologic evaluation for ovarian tumors : Utility in low resource settings and a roadmap for adoption

**DOI:** 10.1016/j.gore.2026.102126

**Published:** 2026-05-27

**Authors:** Aaron Han

**Affiliations:** Loyola University Stritch School of Medicine, Maywood, IL, United States

## Abstract

Intraoperative pathology consultations play an important role in surgical management of oncologic patients. The benefits of frozen section, and cytologic samples are well known and studied. We reviewed the literature and describe a case that highlights some of the advantages and limitations of the cytologic method. We propose a step-wise approach to adopt cytology as the primary method for intraoperative assessment in low resource settings.

## Highlights

• Intraoperative pathology assessment of ovarian tumors is important to guide surgery;
• Frozen section is the gold standard for intraoperative assessment, but is resource intensive;
• Intraoperative cytology may offer low resource settings an alternative in certain situations.

## 1 Introduction

The intraoperative consult is important for guiding appropriate surgical procedures in oncologic settings (Diwagar and Sekhar, 2019, Zarbo et al 1996). In gynecologic oncology, proper intraoperative staging of ovarian tumors is dependent on pathology consultation and interpretation (Begum et al 2023, Huang et al., 2018, Kim, 2019). Sentinel nodes are also important in certain settings, although in gynecologic oncology the utilization of intraoperative sentinel node assessment is not as prevalent as in breast cancer surgery. Uterine cancer may also require intraoperative assessment to confirm grading, and extent of tumor involvement.

Intraoperative frozen section (FS) is well established for ovarian tumors (Hashmi et al 2016, Kim 2019, Naz et al 2016, Shen et al 2021). The purpose can include classification of tumor (benign vs borderline vs malignant), classify tumor type (epithelial, stromal, sarcoma, metastatic), and assess metastatic disease. The accuracy for frozen section diagnosis can be > 95%, with negative predictive value approaching 100%. Discrepancies between frozen section final diagnosis tend to be due to adequacy of tumor sampling, and/or interpretation error (Naz et al 2016, Shen et al 2021).

Intraoperative FS is a resource intensive service, requiring capital equipment and competent technical staff, and trained pathologist. In low resource settings, the lack of equipment (cryostat), technical and interpretative expertise may limit the availability of intraoperative frozen section assessment. Touch prep cytology may be a suitable method in certain contexts and the appropriate types of cases (Ahsan et al., 2025, Ivan et al., 2020). There is literature that suggests touch prep can improve performance in turn-around time (TAT), and the sensitivity and specificity approach frozen section performance (Early et al 2025, Sushma and Panicker 2015).

Prior studies also demonstrated good correlation of TP with FS. Early et al (2025) showed concordance of TP vs FS at 67% vs 80% compared to final histopathology. Turnaround time (TAT) for a single sample of frozen tissue can be performed in under 20 min (Novis and Zarbo 1997). Touch prep can be obtained within 5 min time. Thus, in low resource areas where access to cryostat is limited, touch prep assessment is an alternative to guide the surgeon in their operative approach. One setting in which this issue has surfaced is in International Gynecologic Cancer Society (IGCS) Project ECHO (Extension for Community Healthcare Outcomes), a telementoring program with virtual tumor boards (Randall et al 2021). Not infrequently, surgical procedures are completed at ECHO sites typically a central referral hospital able to support gynecologic oncology with local and international mentors, but without any intraoperative assessment by pathology (Plotkin et al 2019). Most of the limitation for frozen section is the lack of capital equipment. However, lack of trained personnel to prepare a frozen section, or availability of pathologist is also a concern (Bychkov and Fukuoka 2022, Sayed et al 2015, Walsh and Orsi 2024).

In our mini-review we describe a case where touch prep provided timely diagnosis with information to help triage the case and guide the surgeon. We explore the limitation of this approach as a stand-alone procedure, review relevant literature, and discuss how we could implement training to deploy in low-resource settings where cryostat and/or personnel is not available to provide a frozen section consultation service.

## 2 Interesting case

Our example was a 28 year-old patient with abdominal pain and enlarging ovary mass. The non-pregnant patient had enlarged uterine/pelvic area consistent with 32-week fundal height. Imaging showed a 23-cm cystic, septated left ovarian mass. Serum CA-125 was elevated at 289 U/mL (normal < 36), and AFP, β-HCG, inhibin, and LDH were all within normal range.

Intraoperative findings included normal appearing external genitalia, vagina, and cervix. The upper abdomen was free of disease. A large adnexal mass originating from the left ovarian measured 20 cm. Ovary contained mucinous fluid with tissue concerning for malignancy. Bilateral ovaries with adhesions to the posterior *cul-de-sac,* but right ovary and fallopian tube otherwise normal in appearance. Dense adhesions between the bladder and uterus were seen. Omentum with a small nodule was observed, and concern for metastatic disease was raised. No obviously enlarged or positive appearing pelvic or *para*-aortic lymph nodes.

Intraoperative consultation was performed on the left tube and ovary, and separately the omental nodule was sent.

**Fig. 1:**
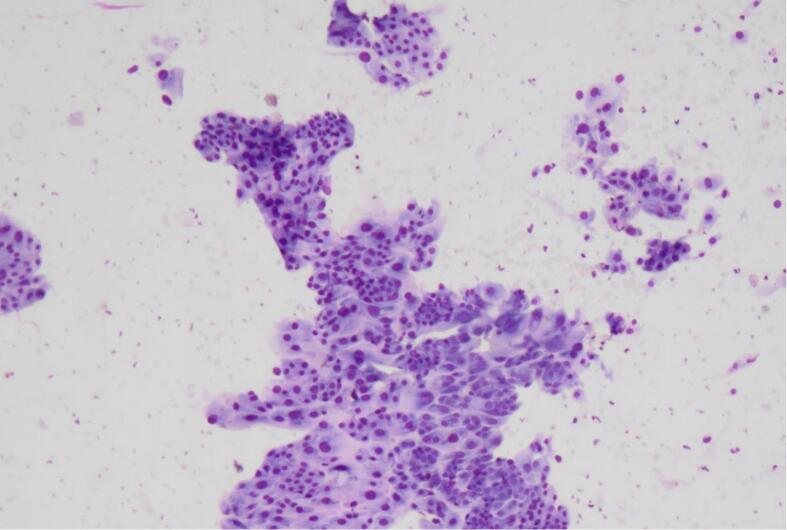
Touch prep shows cohesive epithelial groups. Focal areas of nuclear enlargement and moderate to marked cytologic atypia seen. These were suspicious for focal clear cell differentiation (Diff Quik stain, 10x magnification).

**Fig. 2:**
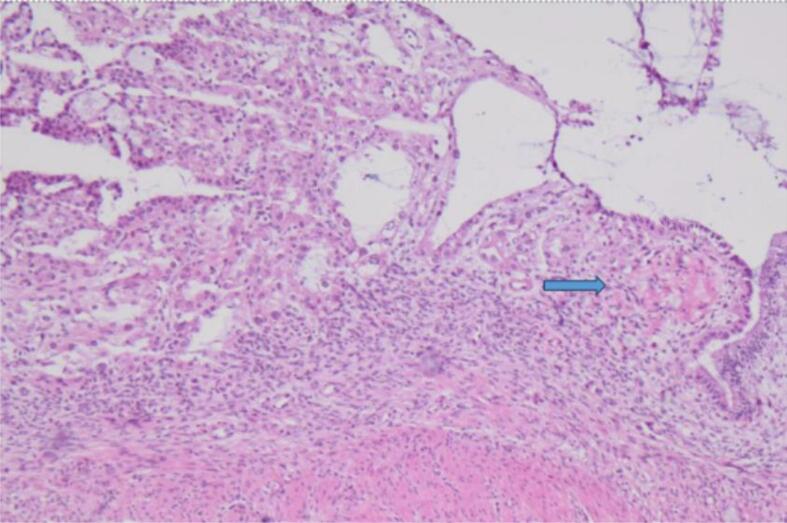
Atypical surface epithelium seen on the frozen section slide. The right side glands had some areas suggestive for endometriosis. Left side of the image shows focal cytologic atypia and area of pink hyaline appear stroma (arrow), suspicious for clear cell features. (H&E stained, 10x magnification). (For interpretation of the references to colour in this figure legend, the reader is referred to the web version of this article.)

**Fig. 3:**
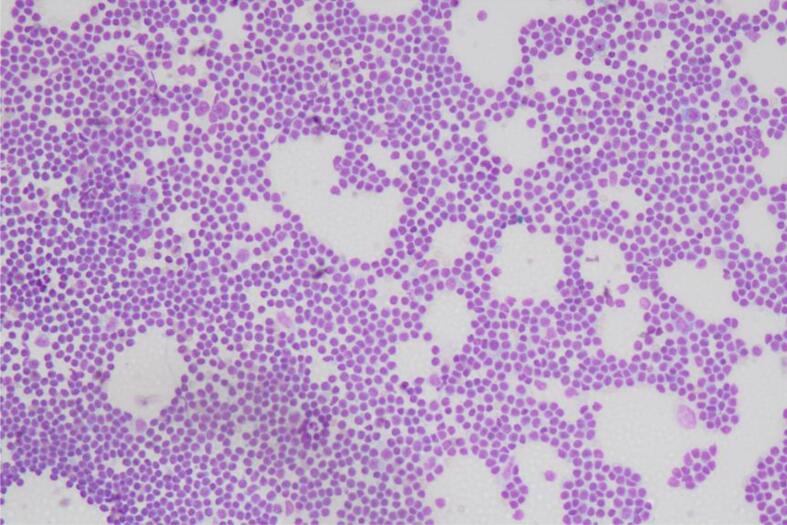
Dishesive groups of small round lymphocytes. Consistent with lymph node from omental nodule touch prep. Stained with Diff Quik (10x magnification).

At gross examination, the ovary had a smooth capsular surface, with no evidence of rupture. The lining of the ovary was predominantly cystic with brown viscid content. Approximately 30% of the ovary was lined by solid areas, with vague papillae. Touch prep sampling the solid area of the ovarian tumor showed epithelial groups with atypia and focal enlargement (Fig. 1). The background showed some degenerated debris, suggestive for tumor diathesis. Frozen section reviewed 5 min later showed borderline changes and one focus of possible hobnailing, and eosinophilic hyaline stroma (Fig. 2). The diagnosis rendered was “At least borderline tumor, rule out clear cell carcinoma.” A second intraoperative consult was requested on the omental nodule to exclude metastatic disease. The touch prep of a nodular tan soft tissue fragment showed non-cohesive small cells consistent with reactive lymphocytes (Fig. 3). The frozen section confirmed the diagnosis of a reactive lymph node.

Final pathology was seromucinous borderline tumor, and the focal clear cell areas were inconclusive even on more extensive tissue sampling and immunohistochemical analysis (tumor was napsin and ER positive). A second opinion of the final pathology concurred with the diagnosis of borderline tumor without evidence of frank clear cell carcinoma or invasion.

## 3 Discussion

Our case illustrates the utility of intraoperative touch preparation in the surgical management of ovarian tumors. Prior studies (Early et al 2025) showed high degree of concordance of intraoperative touch prep vs frozen section when compared to final sign-out results. In our case, we see some of the limitations of touch prep especially with borderline tumors, and there will be a subset of cases that malignancy cannot be confirmed/excluded by touch prep. Also, we are not advocating using cytologic diagnosis to replace histologic exam for the final pathology report. Cytologic exam in gynecologic cancer cases are limited typically to fluid cytology. A prior report from Nepal (Vaidya et al 2020) showed in their series of 101 cases comparing cytology versus final histopathology, majority of their cases were younger in age (93% under 50 years old) with 3 of 11 malignant cases (where the age of patient was known) presenting in patients over 50 years of age. The vast majority of their patients showed benign histology (85%), with four borderline tumors, and 12 malignant tumors. Processing time was 20-minutes based on hematoxylin and eosin type of procedure. They analyzed their cases with a two-tiered benign vs malignant classification. The report shows that intraoperative cytology is feasible in their settings. Improvement with turn-around time can be achieved by using a rapid cytologic staining preparation. Nagai et al (2001) shows good concordance for benign and malignant tumors, but low agreement for borderline tumor cytology diagnosis, rendered intraoperatively, compared to final pathology (30%). Ivan et al (2020), and other authors did not diagnose borderline tumors and cited this as a limitation for cytologic evaluation. This is not surprising, as even with frozen section analysis, and an average of 3 sections per case, borderline tumors cannot be accurately determined on frozen section analysis, with the highest discrepancies seen in mucinous and unilateral tumors (Huang et al 2018). Abe et al (2013) examined germ cell tumors, and immature teratomas could not be reliably discerned on cytology. Likely these cases are limited by sampling, and ability to reliably sample immature elements or the percentage of those elements is low.

Sushma and Panicker (2015) reported their series from India with a total of 195 cases, 186 cases were classified by intraoperative cytology into epithelial (n = 122), stromal (n = 8), germ cell (n = 25), and other (n = 31), with accuracy of 96%, 98%, 100%, 97%, respectively.

### 3.1 Challenges in classification of tumor

When a tumor cannot be definitively classified as malignant, the gross examination may be a useful guide in some situations. The recommendation is for indeterminate cases, they be treated as cancers with a more extensive staging procedure (Ahsan et al 2025).

Epithelial vs stromal vs lymphoid lesions can be readily discerned by defined cytologic features. Epithelial and stromal tumors also have distinct gross appearances, where together with touch preps, one can reliably classify tumors without histologic frozen sections (Kim 2019).

Primary vs metastatic tumors can be the focus of an intraoperative consultation. Cytologic features such as Krukenberg tumors with garland type of tumor cell arrangements, and background of dirty necrosis; single filing features of metastatic lobular carcinomas of the breast with cytoplasmic vacuoles; pigmented cells in melanoma can be suspected in the appropriate settings (Kim 2019).

In low resource setting where no cryostat is available, we believe that intraoperative consultation in the form of gross examination and imprint or scrape cytology can add value. Our case and the literature highlights the settings in which the cytologic findings can add information for surgery, and the limitation when a borderline tumor is in the differential (which can be the case with frozen sections and needing adequate and extensive sampling to address in final pathologic exam).

### 3.2 Risk based approach

Shen et al (2021) reviewed the literature to determine factors leading to discrepant diagnoses between intraoperative and final pathology. Younger patient age (under 50), low tumor stage, mucinous and endometrioid histologic type were associated with non-concordance. Independent multivariate analysis showed that menopause patients, multicystic tumor features on ultrasound, and the presence of ascites were also factors that led to non-agreement (Shen et al 2021). One should consider these factors that have been identified in the literature, but also review internal institutional data and statistics to improve performance when discrepant results occur. Classifying cases of non-concordance into minor or major discrepancies based on clinical impact, and perform a proper root cause analysis when major discrepancies occur (Novis 2004). Begum et al (2023), showed that insufficient tissue sampling and less than satisfactory technique and frozen section artifacts were the most frequent causes for major discrepancies leading to inadequate surgery and staging.

### 3.3 Other applications in gynecologic oncology surgery

We had a recent hysterectomy case submitted for intraoperative consultation, the absence of a tumor or polypoid lesion in the corpus with a negative scraping cytology is highly correlative with a negative final uterine corpus pathology. The uterine serous tumor phenotype can be detected or suspected in cases where a gross lesion is not seen. In these cases, touch prep likely can sample a larger proportion of the uterine surface compared to frozen sections. Examination of the cervix for invasion can be assessed using touch prep as well. These are areas of application that would be worth examining in future studies and correlation of results with final pathology.

### 3.4 Proposed roadmap and training curriculum

We suggest a three-step approach to train pathologists on the use of intraoperative touch preps for consultation in low resource settings. The first would be pathologist interpretation training on glass or digital slides (static images or whole slide images WSI) with representative tumor types. The focus would be recognition of epithelial vs stromal vs germ cell tumors. One would assess the competency using a standard set of images for testing.

Training of technical staff and/or pathologist on taking cytologic specimens would be included in the preanalytical portion of training. Smear types would include imprints, and scrapes. Adequate and extensive sampling of multiloculated and large tumors would be part of the curriculum. One can do simulated training with non-patient specimens, or other cases of fresh patient tissue. The ability to obtain well preserved and adequate numbers of cells on a slide is important to optimize diagnostic yield and minimize limited or indeterminate results.

Following training on images, we suggest that onsite or hybrid proctored training with real cases be performed. This would allow the proctor/mentor to help with solving any technical issues, and interpretative challenges in a “live” setting. This could be preceded by simulated cases with known histories to develop skills at troubleshooting common problems encountered in practice.

Finally, ongoing quality metrics and monitoring of the touch prep service should include benchmarking for turn-around-time, and concordance with final pathology results. These should be followed-up with corrective action to ensure timely and quality results (Laakman et al 2021).

We believe that with proper training and monitoring of the technique, intraoperative touch preps can be useful to supplement frozen section (Abdelghany et al 2015) and in low resource settings where access to cryostats are not available, they provide useful information to guide appropriate surgery.

## CRediT authorship contribution statement

**Aaron Han:** Writing – review & editing, Writing – original draft, Supervision, Methodology, Conceptualization.

## Declaration of competing interest

The authors declare that they have no known competing financial interests or personal relationships that could have appeared to influence the work reported in this paper.
